# Rapid recovery after intrathecal dexamethasone in FIRES

**DOI:** 10.1002/epd2.70173

**Published:** 2026-01-08

**Authors:** João Filipe Nico, Ana Rita Fradique, Catarina Teixeira, Rita Moinho, Mariana Leitão Marques, Constança Santos, Joana Afonso Ribeiro, Filipe Palavra, Cristina Pereira

**Affiliations:** ^1^ Center for Child Development – Neuropediatrics Unit, Hospital Pediátrico Unidade Local de Saúde de Coimbra Coimbra Portugal; ^2^ Pediatric Intensive Care Unit, Hospital Pediátrico Unidade Local de Saúde de Coimbra Coimbra Portugal; ^3^ Faculty of Medicine University of Coimbra Coimbra Portugal; ^4^ Reference Center for Refractory Epilepsy Unidade Local de Saúde de Coimbra and European Reference Network EpiCare Coimbra Portugal

Febrile infection‐related epilepsy syndrome (FIRES) is a rare and severe epileptic encephalopathy affecting previously healthy individuals, with high morbidity and mortality. This condition is defined by new‐onset refractory status epilepticus following a nonspecific febrile illness, without identifiable structural, metabolic, or infectious causes, and frequently progresses to super‐refractory status epilepticus (SRSE).^1^, ^2^ Diagnosis is clinical, requiring an extensive work‐up.^1^, ^2^ Management involves escalation of antiseizure medications (ASMs), early ketogenic diet (KD), and immunotherapy, though responses are often limited.^1^, ^2^, ^3^ Intrathecal dexamethasone (IT‐DEX) has emerged as a potential adjunctive option in refractory cases.^4^, ^5^

We report a previously healthy 15‐year‐old boy with generalized tonic–clonic seizures and fever after 5 days of asthenia, somnolence, and headache. Initial investigations (blood tests, lumbar puncture, cranial computed tomography, and brain and spinal magnetic resonance imaging) showed mild leukocytosis and elevated C‐reactive protein. Video‐electroencephalography (vEEG) demonstrated diffuse slowing with a sparse alpha rhythm.

Empirical acyclovir, ceftriaxone, ciprofloxacin, and methylprednisolone were started for presumed infectious or autoimmune encephalitis. Within 24 h, he progressed to SRSE with respiratory failure, requiring pediatric intensive care admission.

Extensive autoimmune, metabolic, infectious, immunologic, and paraneoplastic work‐up was unremarkable, except for positive immunoglobulin M and immunoglobulin G (IgG) for *Mycoplasma pneumoniae* (recent infection) and IgG for *Listeria monocytogenes*. Whole‐exome sequencing revealed no pathogenic variants.

SRSE persisted despite continuous midazolam and multiple ASMs (levetiracetam, valproic acid, lacosamide, clobazam, perampanel, phenobarbital, and phenytoin), and invasive mechanical ventilation was required on day seven for worsening consciousness.

After a 7‐day course of high‐dose intravenous methylprednisolone with taper, immunotherapy with intravenous immunoglobulin (2 g/kg over 3 days) and therapeutic plasma exchange (five sessions) was ineffective. The KD also failed to control seizures.

On day 12, thiopental achieved burst suppression but caused hemodynamic instability requiring norepinephrine infusion and gastrointestinal intolerance. Following thiopental withdrawal, seizures recurred despite ketamine and cenobamate. With a working diagnosis of FIRES, anakinra (300 mg/day, subcutaneous) was initiated on day 21, yet seizures persisted. The KD was reintroduced on day 26 with good tolerance but limited effect.

By day 37, SRSE persisted despite maximal therapy, prompting IT‐DEX as a late rescue. Each dose was 12 mg (0.15 mg/kg) dexamethasone diluted in sterile saline to a total volume of 5 mL, instilled slowly after withdrawal of an equivalent volume of cerebrospinal fluid (CSF). IT‐DEX was administered for three consecutive days, followed by three additional doses on alternate days. Seizures ceased after the second dose, allowing anesthetic weaning and extubation on day 39. IT‐DEX was well tolerated, with no adverse effects despite the use of a preservative‐containing formulation. The patient progressively recovered neurological function.

CSF obtained before anakinra later showed markedly elevated interleukin‐6 (IL‐6). Given this IL‐6 predominant profile and the limited response to IL‐1 blockade, immunotherapy was switched from anakinra to monthly intravenous tocilizumab (8 mg/kg) to target persistent IL‐6‐driven inflammation despite seizure remission.

He was transferred to the ward on day 53 on multiple ASMs. On day 57, a brief cluster of focal seizures occurred during a febrile urinary tract infection, with no further events. Cognitive screening with the Montreal Cognitive Assessment (score 21/30) demonstrated mild deficits in attention and calculation, prompting cognitive rehabilitation. On day 85, he was discharged seizure‐free, ambulant, and on monthly tocilizumab and ASMs.

The overall clinical and therapeutic course is summarized in Figure 1, with vEEG recordings in Figures S1–S5.

**FIGURE 1:**
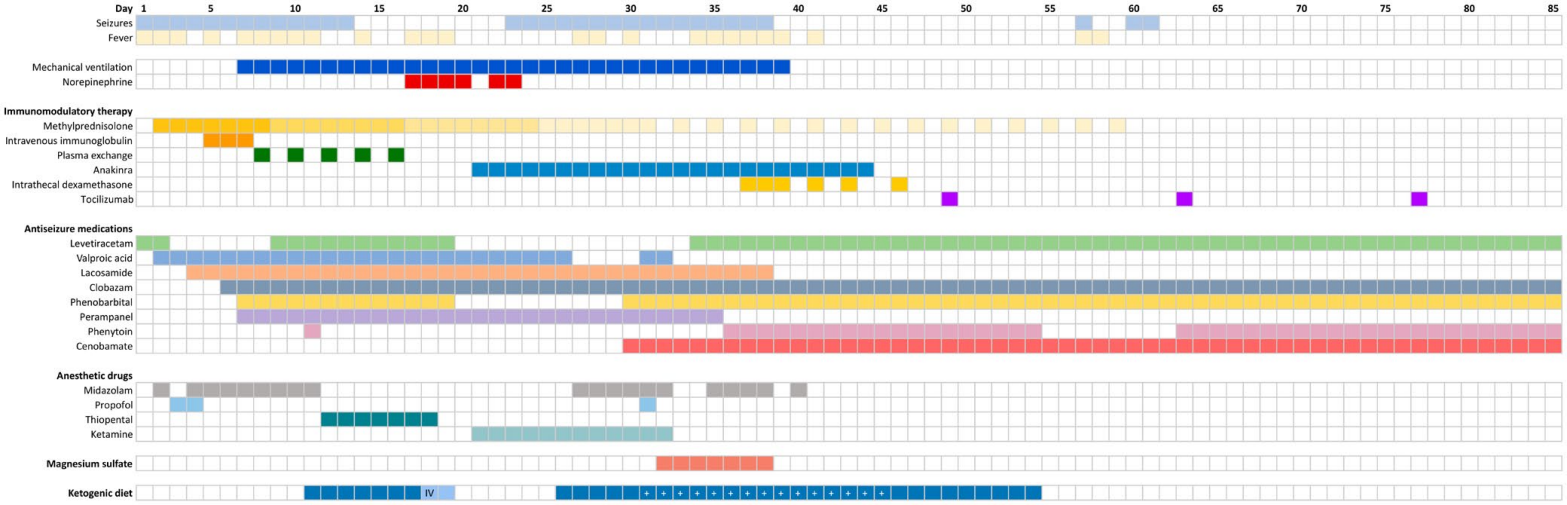
Clinical course during hospitalization. The figure summarizes the patient's hospital course by day (D = day of admission). The upper section illustrates the clinical evolution, including days with seizures and fever. The patient required invasive mechanical ventilation (D7–39) and norepinephrine for cardiovascular support (D17–20, D22–23). Immunomodulatory therapy included methylprednisolone (1000 mg/day, D2–8, with tapering thereafter), intravenous immunoglobulin (2 g/kg, D5–7), and plasma exchange (D8, 10, 12, 14, 16). Subcutaneous anakinra (300 mg/day) was administered from D21–44. Intrathecal dexamethasone was given on D37, 38, 39, 41, 43, and 46 (12 mg/dose, 0.15 mg/kg/dose). Seizures ceased on D39, after the second intrathecal dexamethasone dose. Intravenous tocilizumab (8 mg/kg/dose) was started on D49 and maintained every 15 days, later monthly after discharge, with the plan to complete a six‐month course. Sequential introduction of antiseizure medications (ASMs) and anesthetic agents, titrated to weight and serum levels, is shown. The ketogenic diet was initiated on D11 but not tolerated; intravenous (IV) attempts were unsuccessful. It was resumed on D26, achieving ketosis (2–5 mmol/L, marked as +) from D31 and discontinued on D54. The patient was transferred to the ward on D53. Brief focal seizures recurred on D57 in the setting of a febrile urinary tract infection and shortly after phenytoin discontinuation; phenytoin was reintroduced and seizures resolved with appropriate antimicrobial and antiseizure management. The patient was discharged on D85 under levetiracetam, clobazam, phenobarbital, phenytoin, and cenobamate, continuing physical and cognitive rehabilitation. ASMs were maintained without taper at discharge, with future reduction to be considered only after sustained seizure freedom and consolidation of rehabilitation.

Recent cohorts report around 15%–25% mortality and frequent long‐term cognitive impairment and drug‐resistant epilepsy.^3^, ^6^, ^7^, ^8^, ^9^ Early recognition and escalation to immunotherapy are recommended, although responses remain inconsistent.^1^, ^2^, ^3^, ^6^, ^7^ The complexity of FIRES may preclude strict adherence to recommendations, as illustrated by delayed anakinra initiation in this case.

IT‐DEX has been used as a late rescue after failure of multiple immunotherapies and anesthetic regimens, with seizure improvement often occurring within a few doses and no reported adverse events.^4^, ^5^ This pattern is consistent with the temporal course observed in our patient, in whom seizures ceased after the second IT‐DEX dose. The rapid seizure cessation supports direct intrathecal corticosteroid modulation of central nervous system inflammation and may more effectively downregulate pro‐inflammatory cytokine cascades than systemic steroids alone.^3^, ^4^, ^8^, ^9^

In summary, IT‐DEX may represent a biologically plausible adjunctive rescue therapy for super‐refractory FIRES, offering rapid seizure control and neurological recovery.

## CONFLICT OF INTEREST STATEMENT

None of the authors has any conflict of interest to disclose.

## PATIENT CONSENT STATEMENT

Written informed consent for publication of the clinical details and accompanying images included in this report was obtained from the patient's legal guardians.

**Test Yourself**

1. What is the main proposed mechanism of action of intrathecal dexamethasone in FIRES?
  - Enhancement of GABAergic transmission.
  - Reduction of neuronal excitability through sodium channel blockade.
  - Direct anti‐inflammatory effect within the CNS reducing cytokine‐mediated neuroinflammation.
  - Increased cerebral blood flow to affected regions.
  - Modulation of synaptic glutamate release.
2. What was the observed clinical response following intrathecal dexamethasone in this patient with FIRES?
  - No change in seizure frequency despite multiple doses.
  - Seizure cessation after the second intrathecal dose and progressive neurological recovery.
  - Initial improvement followed by relapse 2 weeks later.
  - Persistent super‐refractory status epilepticus requiring long‐term anesthetic therapy.
  - Transient improvement associated with significant adverse effects.
3. Which statement best reflects current understanding of FIRES management?
  - FIRES typically resolves spontaneously without immunotherapy.
  - The ketogenic diet consistently leads to seizure control.
  - Early recognition and initiation of immunotherapy are crucial, although therapeutic responses remain inconsistent.
  - Mortality rates are generally below 5%.
  - Long‐term cognitive outcomes are usually unaffected.

*Answers may be found in the* *Supporting information*.

## Supporting information


Data S1

Data S2

Figures S1‐S5

## Data Availability

The data that support the findings of this study are available on request from the corresponding author. The data are not publicly available due to privacy or ethical restrictions.
