# Supplementary material for: Rapid recovery after intrathecal dexamethasone in FIRES
Source: Epileptic Disord. 2026 Jan 8;28(3):884–7. doi: 10.1002/epd2.70173 (PMC13276701; doi:10.1002/epd2.70173)
Supplement: Supplementary file 1 — Data S1 [file EPD2-28-884-s001.docx]

**TEST YOURSELF ANSWER**

**Answer 1:**

C) Direct anti-inflammatory effect within the CNS reducing cytokine-mediated neuroinflammation

Rationale:

Intrathecal dexamethasone is believed to exert a direct anti-inflammatory effect within the central nervous system, reducing cytokine-driven neuroinflammation not controlled by systemic therapy. Other options describe unrelated or less specific mechanisms.

**Answer 2:**

B) Seizure cessation after the second intrathecal dose and progressive neurological recovery

Rationale:

The patient experienced complete seizure cessation after the second intrathecal dose, enabling anesthetic weaning and extubation, with subsequent steady neurological recovery and no complications.

**Answer 3:**

C) Early recognition and initiation of immunotherapy are crucial, although therapeutic responses remain inconsistent

Rationale:

FIRES is a severe, treatment-resistant epileptic encephalopathy. Early initiation of immunotherapy is essential, but responses vary widely. Mortality can reach 15–25%, and long-term cognitive impairment is frequent.
