# Supplementary material for: Rapid recovery after intrathecal dexamethasone in FIRES
Source: Epileptic Disord. 2026 Jan 8;28(3):884–7. doi: 10.1002/epd2.70173 (PMC13276701; doi:10.1002/epd2.70173)
Supplement: Supplementary file 2 — Data S2 [file EPD2-28-884-s003.pptx]

## Slide 1
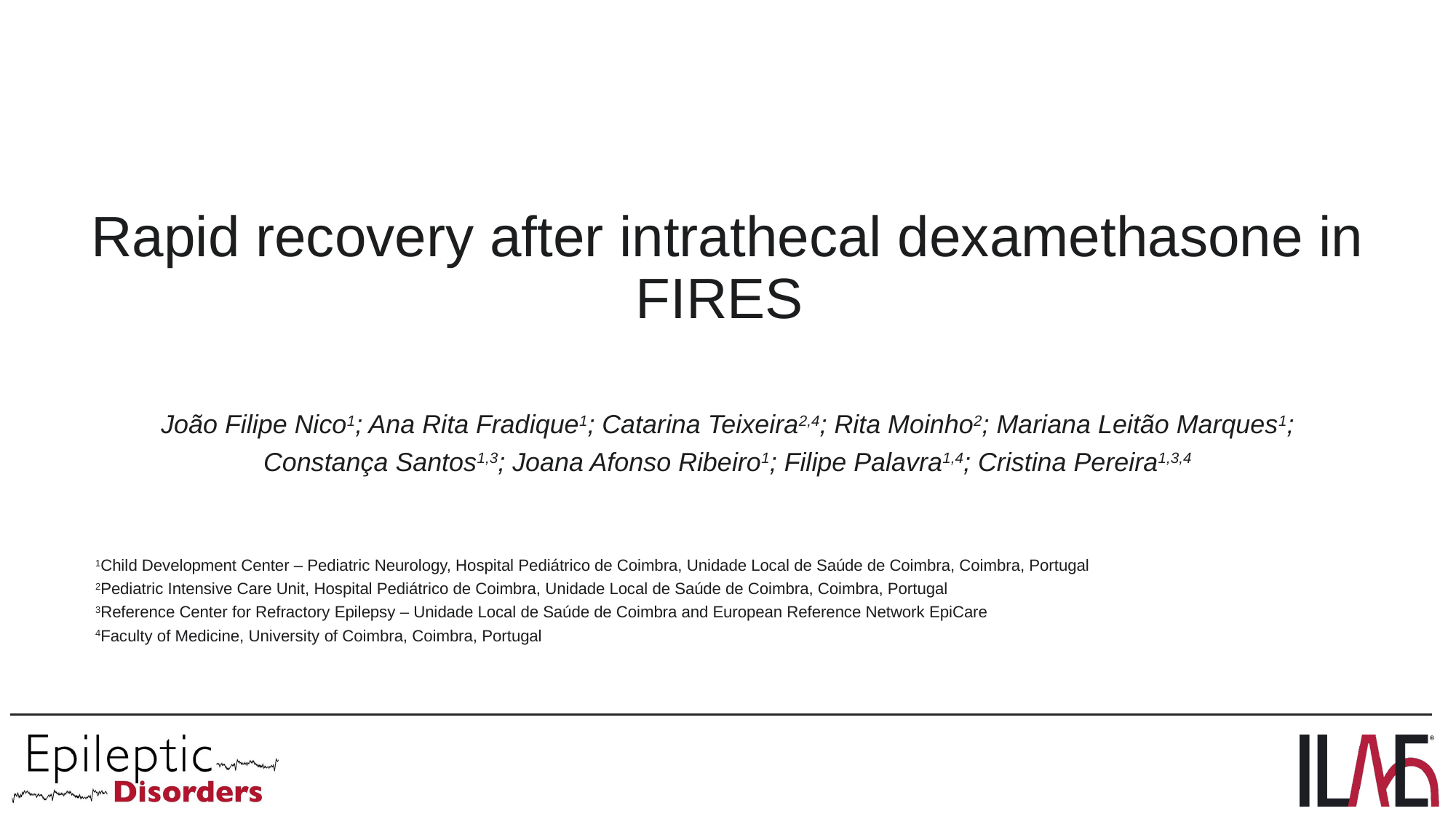

# Rapid recovery after intrathecal dexamethasone in FIRES
João Filipe Nico1; Ana Rita Fradique1; Catarina Teixeira2,4; Rita Moinho2; Mariana Leitão Marques1; Constança Santos1,3; Joana Afonso Ribeiro1; Filipe Palavra1,4; Cristina Pereira1,3,4
1Child Development Center – Pediatric Neurology, Hospital Pediátrico de Coimbra, Unidade Local de Saúde de Coimbra, Coimbra, Portugal
2Pediatric Intensive Care Unit, Hospital Pediátrico de Coimbra, Unidade Local de Saúde de Coimbra, Coimbra, Portugal
3Reference Center for Refractory Epilepsy – Unidade Local de Saúde de Coimbra and European Reference Network EpiCare
4Faculty of Medicine, University of Coimbra, Coimbra, Portugal

## Slide 2
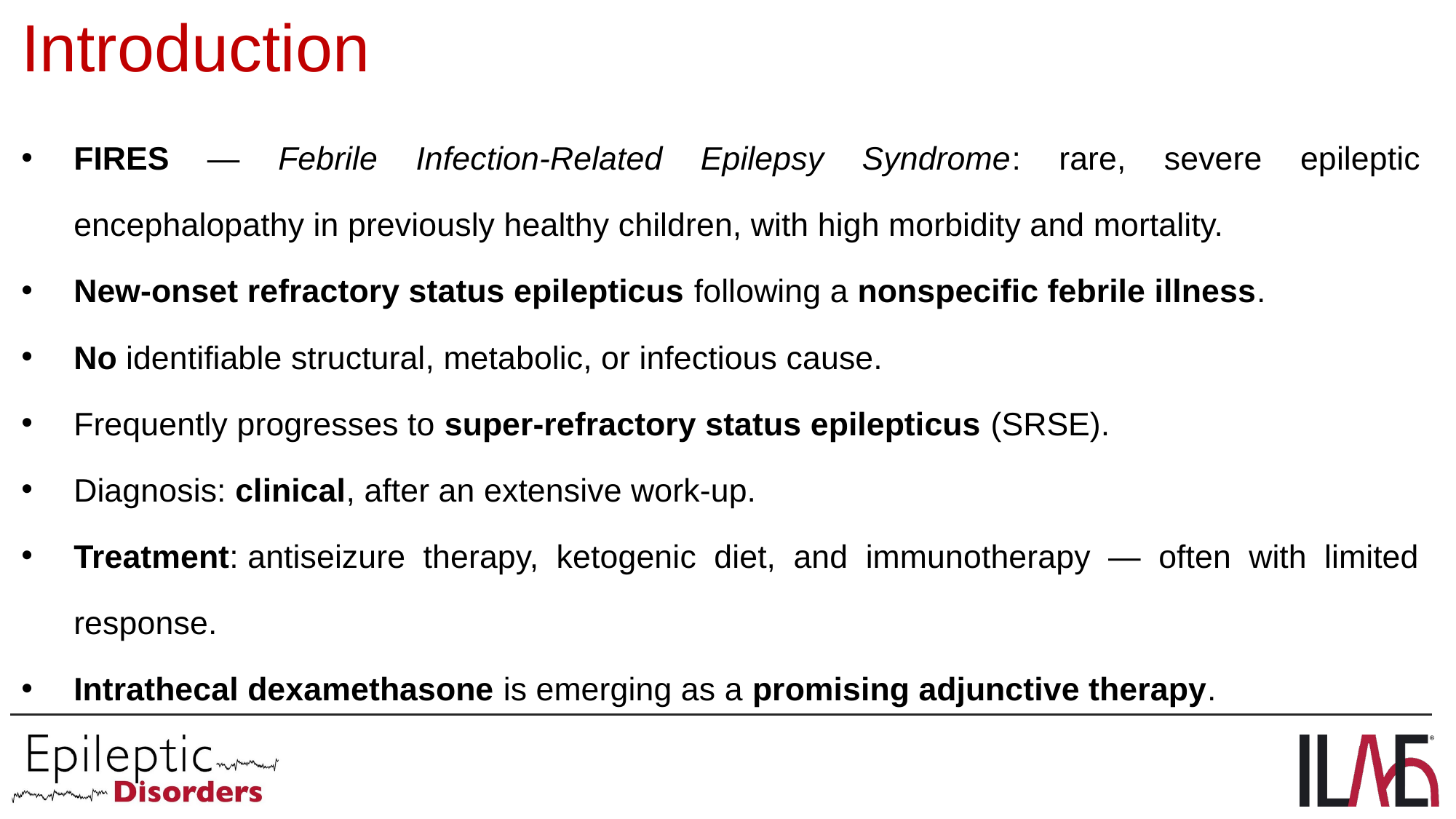

Introduction
FIRES — Febrile Infection-Related Epilepsy Syndrome: rare, severe epileptic encephalopathy in previously healthy children, with high morbidity and mortality.
New-onset refractory status epilepticus following a nonspecific febrile illness.
No identifiable structural, metabolic, or infectious cause.
Frequently progresses to super-refractory status epilepticus (SRSE).
Diagnosis: clinical, after an extensive work-up.
Treatment: antiseizure therapy, ketogenic diet, and immunotherapy — often with limited response.
Intrathecal dexamethasone is emerging as a promising adjunctive therapy.

## Slide 3
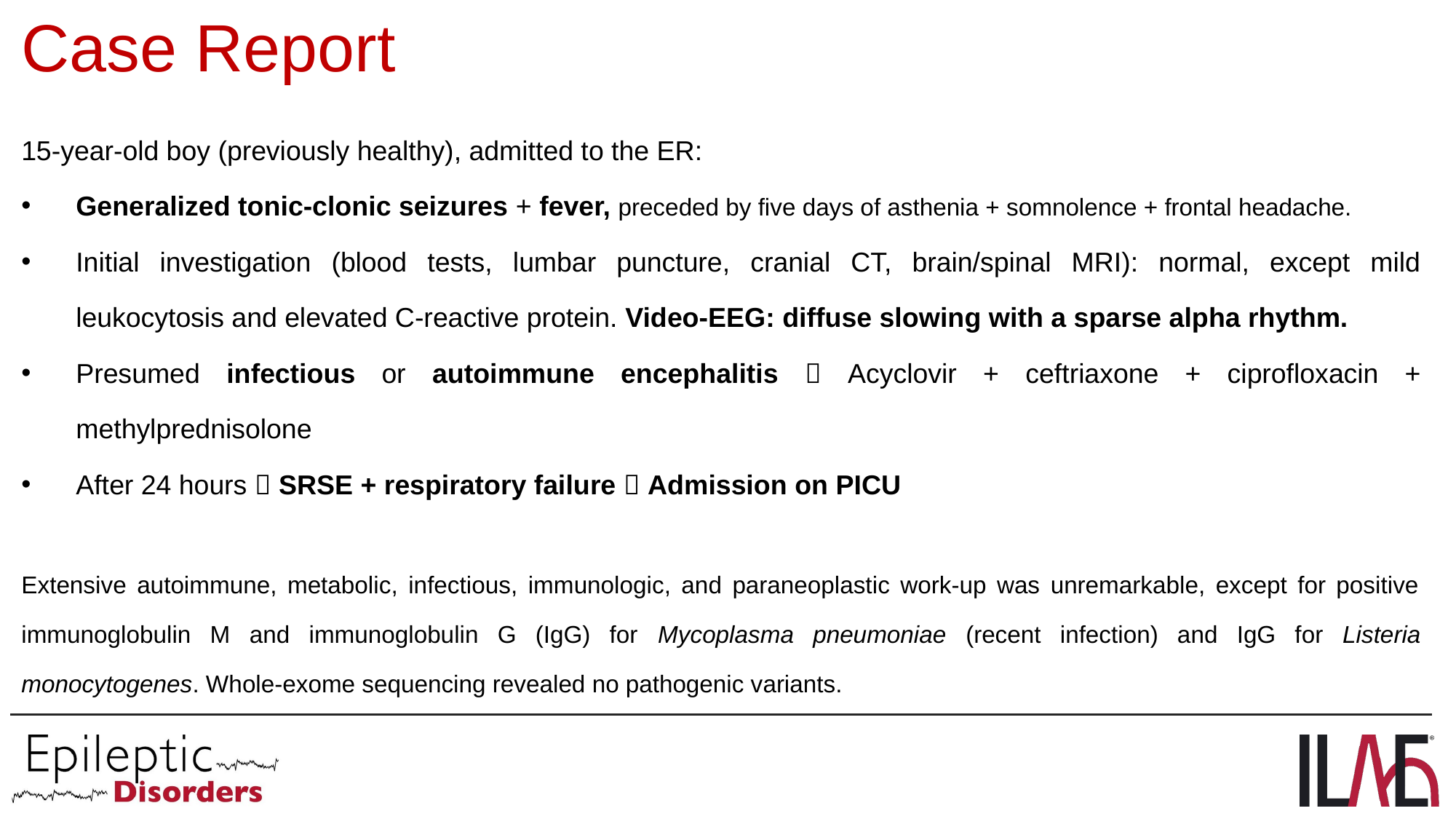

Case Report
15-year-old boy (previously healthy), admitted to the ER:
Generalized tonic-clonic seizures + fever, preceded by five days of asthenia + somnolence + frontal headache.
Initial investigation (blood tests, lumbar puncture, cranial CT, brain/spinal MRI): normal, except mild leukocytosis and elevated C-reactive protein. Video-EEG: diffuse slowing with a sparse alpha rhythm.
Presumed infectious or autoimmune encephalitis  Acyclovir + ceftriaxone + ciprofloxacin + methylprednisolone
After 24 hours  SRSE + respiratory failure  Admission on PICU
Extensive autoimmune, metabolic, infectious, immunologic, and paraneoplastic work-up was unremarkable, except for positive immunoglobulin M and immunoglobulin G (IgG) for Mycoplasma pneumoniae (recent infection) and IgG for Listeria monocytogenes. Whole-exome sequencing revealed no pathogenic variants.

## Slide 4
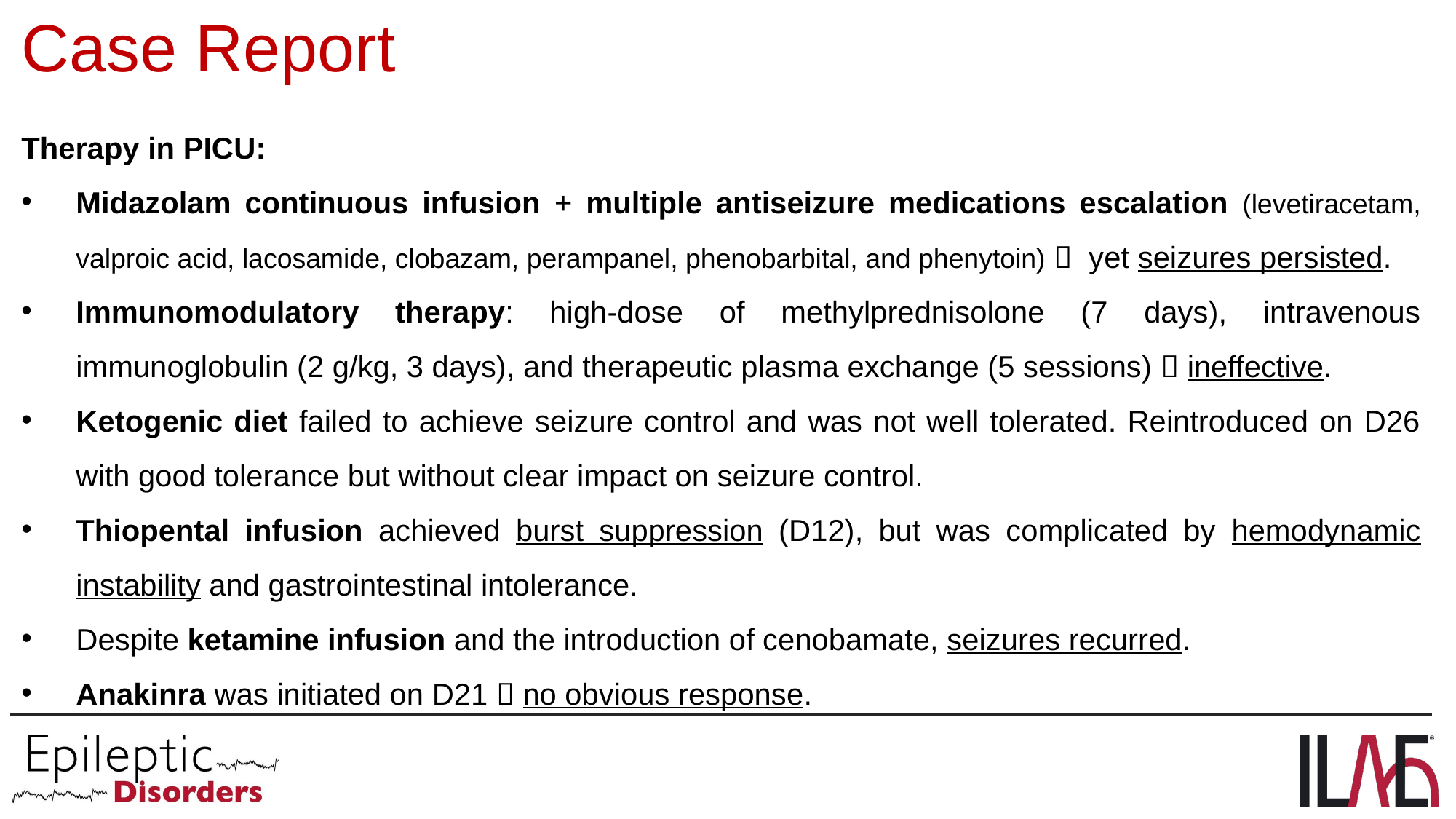

Case Report
Therapy in PICU:
Midazolam continuous infusion + multiple antiseizure medications escalation (levetiracetam, valproic acid, lacosamide, clobazam, perampanel, phenobarbital, and phenytoin)  yet seizures persisted.
Immunomodulatory therapy: high-dose of methylprednisolone (7 days), intravenous immunoglobulin (2 g/kg, 3 days), and therapeutic plasma exchange (5 sessions)  ineffective.
Ketogenic diet failed to achieve seizure control and was not well tolerated. Reintroduced on D26 with good tolerance but without clear impact on seizure control.
Thiopental infusion achieved burst suppression (D12), but was complicated by hemodynamic instability and gastrointestinal intolerance.
Despite ketamine infusion and the introduction of cenobamate, seizures recurred.
Anakinra was initiated on D21  no obvious response.

## Slide 5
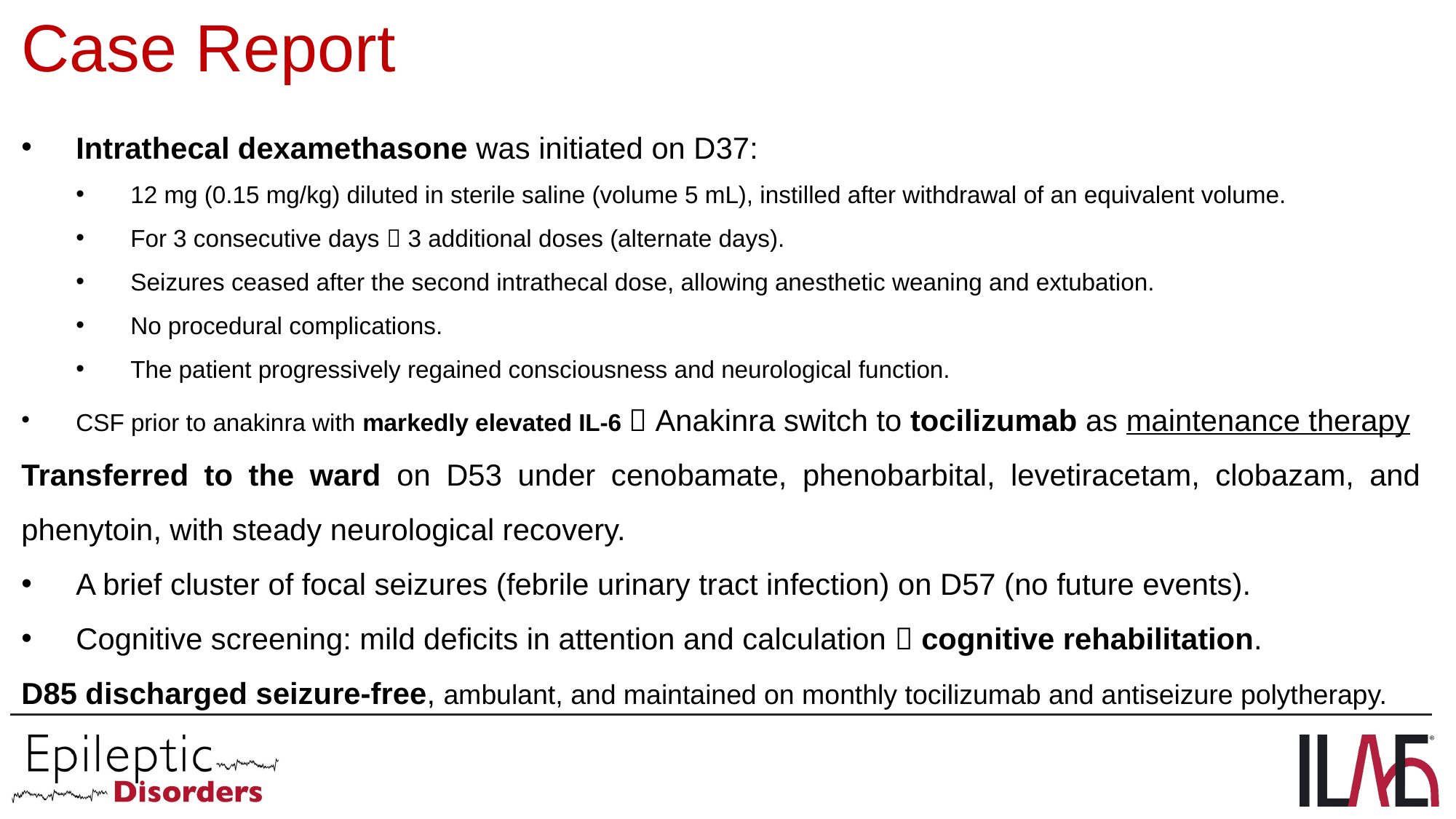

Case Report
Intrathecal dexamethasone was initiated on D37:
12 mg (0.15 mg/kg) diluted in sterile saline (volume 5 mL), instilled after withdrawal of an equivalent volume.
For 3 consecutive days  3 additional doses (alternate days).
Seizures ceased after the second intrathecal dose, allowing anesthetic weaning and extubation.
No procedural complications.
The patient progressively regained consciousness and neurological function.
CSF prior to anakinra with markedly elevated IL-6  Anakinra switch to tocilizumab as maintenance therapy
Transferred to the ward on D53 under cenobamate, phenobarbital, levetiracetam, clobazam, and phenytoin, with steady neurological recovery.
A brief cluster of focal seizures (febrile urinary tract infection) on D57 (no future events).
Cognitive screening: mild deficits in attention and calculation  cognitive rehabilitation.
D85 discharged seizure-free, ambulant, and maintained on monthly tocilizumab and antiseizure polytherapy.

## Slide 6
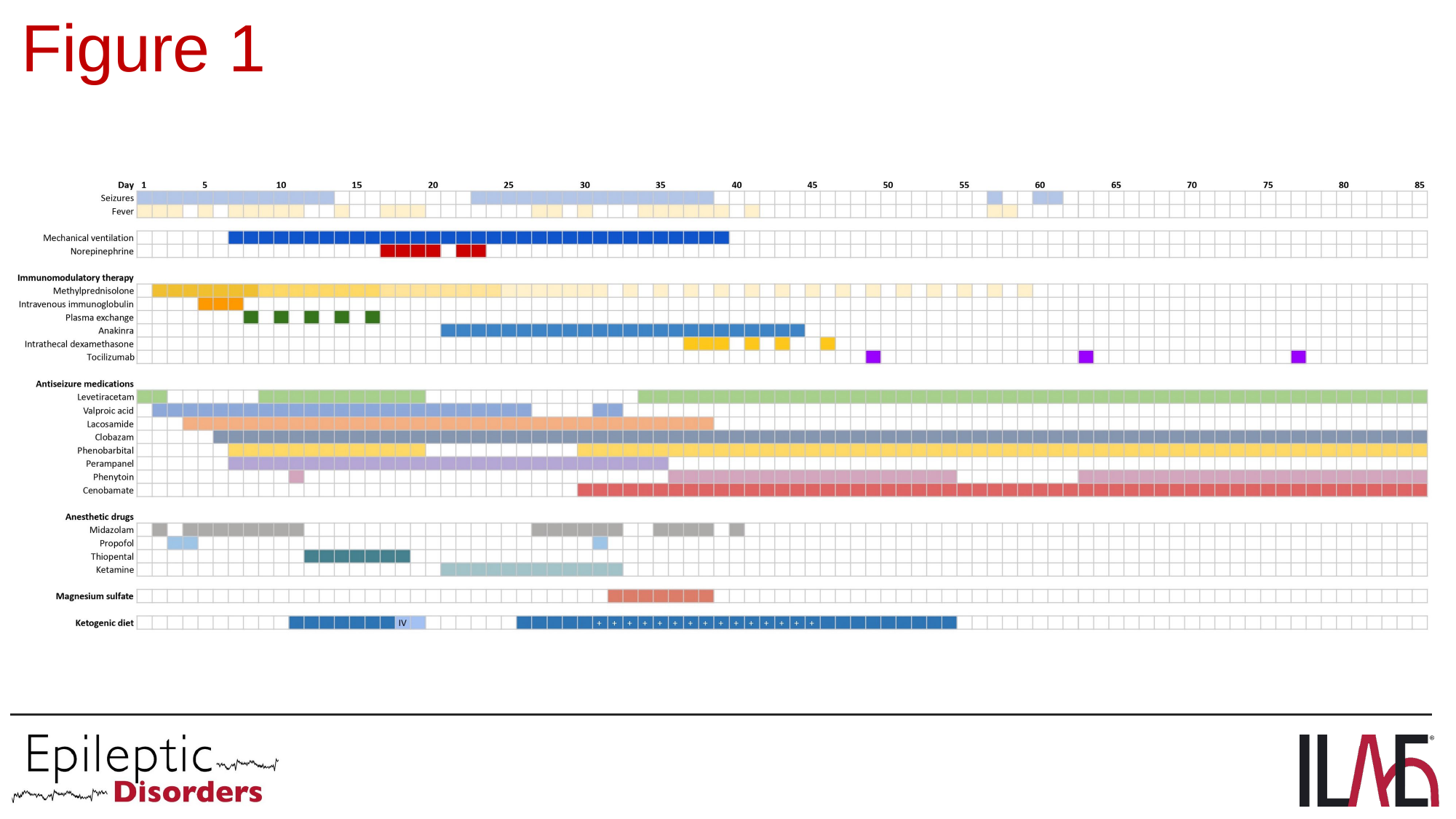

Figure 1

## Slide 7
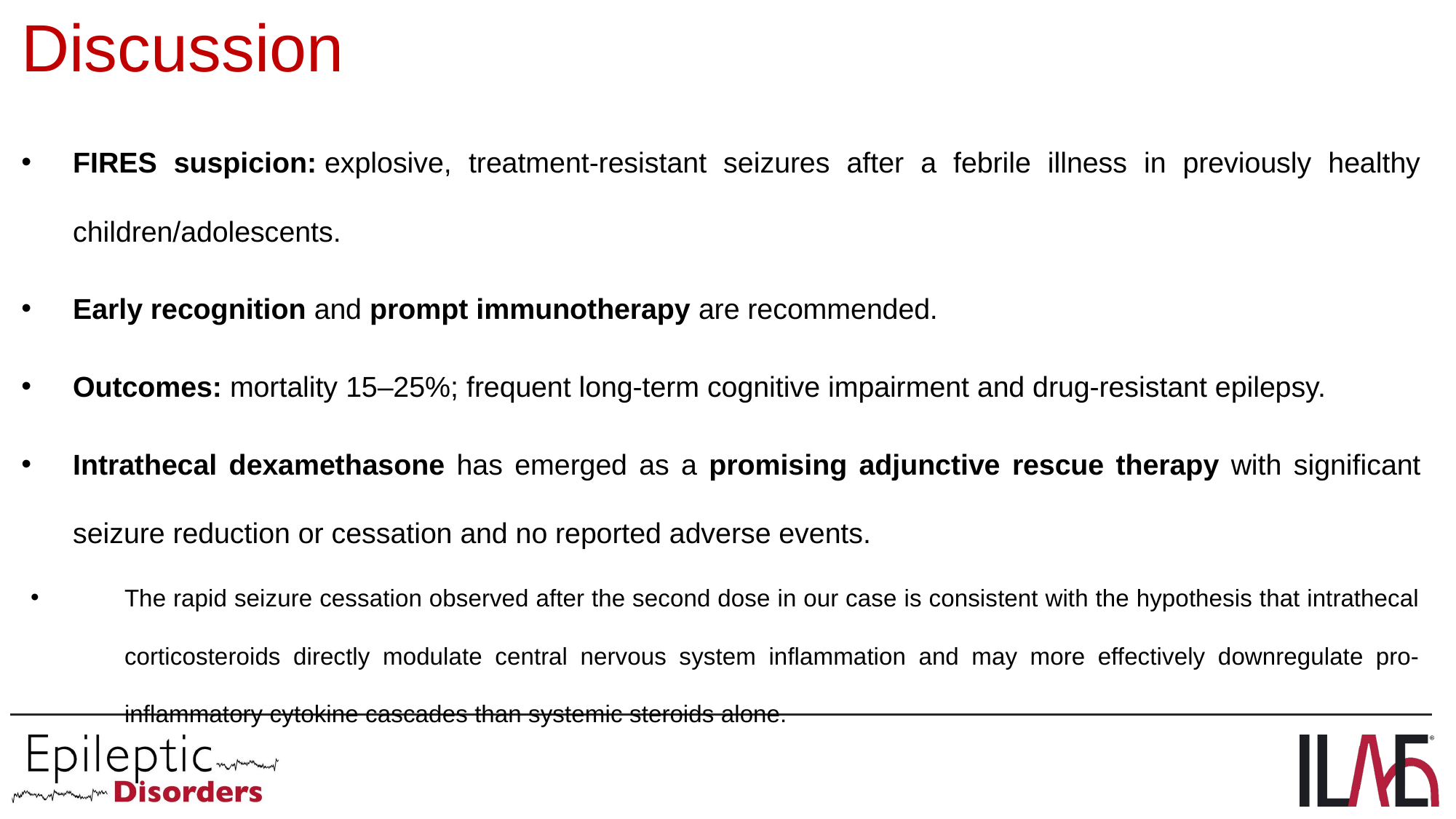

Discussion
FIRES suspicion: explosive, treatment-resistant seizures after a febrile illness in previously healthy children/adolescents.
Early recognition and prompt immunotherapy are recommended.
Outcomes: mortality 15–25%; frequent long-term cognitive impairment and drug-resistant epilepsy.
Intrathecal dexamethasone has emerged as a promising adjunctive rescue therapy with significant seizure reduction or cessation and no reported adverse events.
The rapid seizure cessation observed after the second dose in our case is consistent with the hypothesis that intrathecal corticosteroids directly modulate central nervous system inflammation and may more effectively downregulate pro-inflammatory cytokine cascades than systemic steroids alone.

## Slide 8
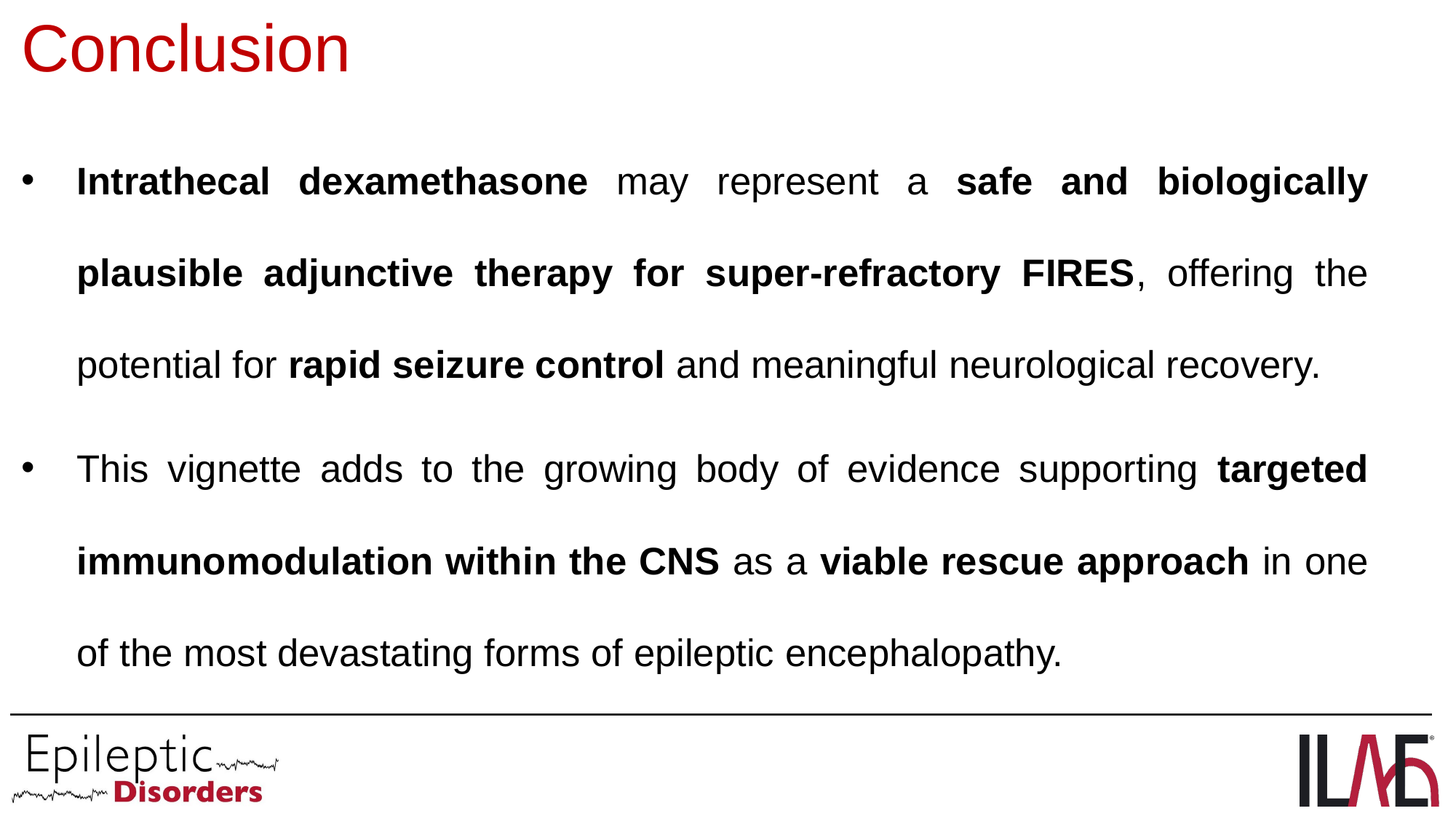

Conclusion
Intrathecal dexamethasone may represent a safe and biologically plausible adjunctive therapy for super-refractory FIRES, offering the potential for rapid seizure control and meaningful neurological recovery.
This vignette adds to the growing body of evidence supporting targeted immunomodulation within the CNS as a viable rescue approach in one of the most devastating forms of epileptic encephalopathy.
