# Supplementary material for: Rapid recovery after intrathecal dexamethasone in FIRES
Source: Epileptic Disord. 2026 Jan 8;28(3):884–7. doi: 10.1002/epd2.70173 (PMC13276701; doi:10.1002/epd2.70173)
Supplement: Supplementary file 3 — Figures S1‐S5 [file EPD2-28-884-s002.docx]

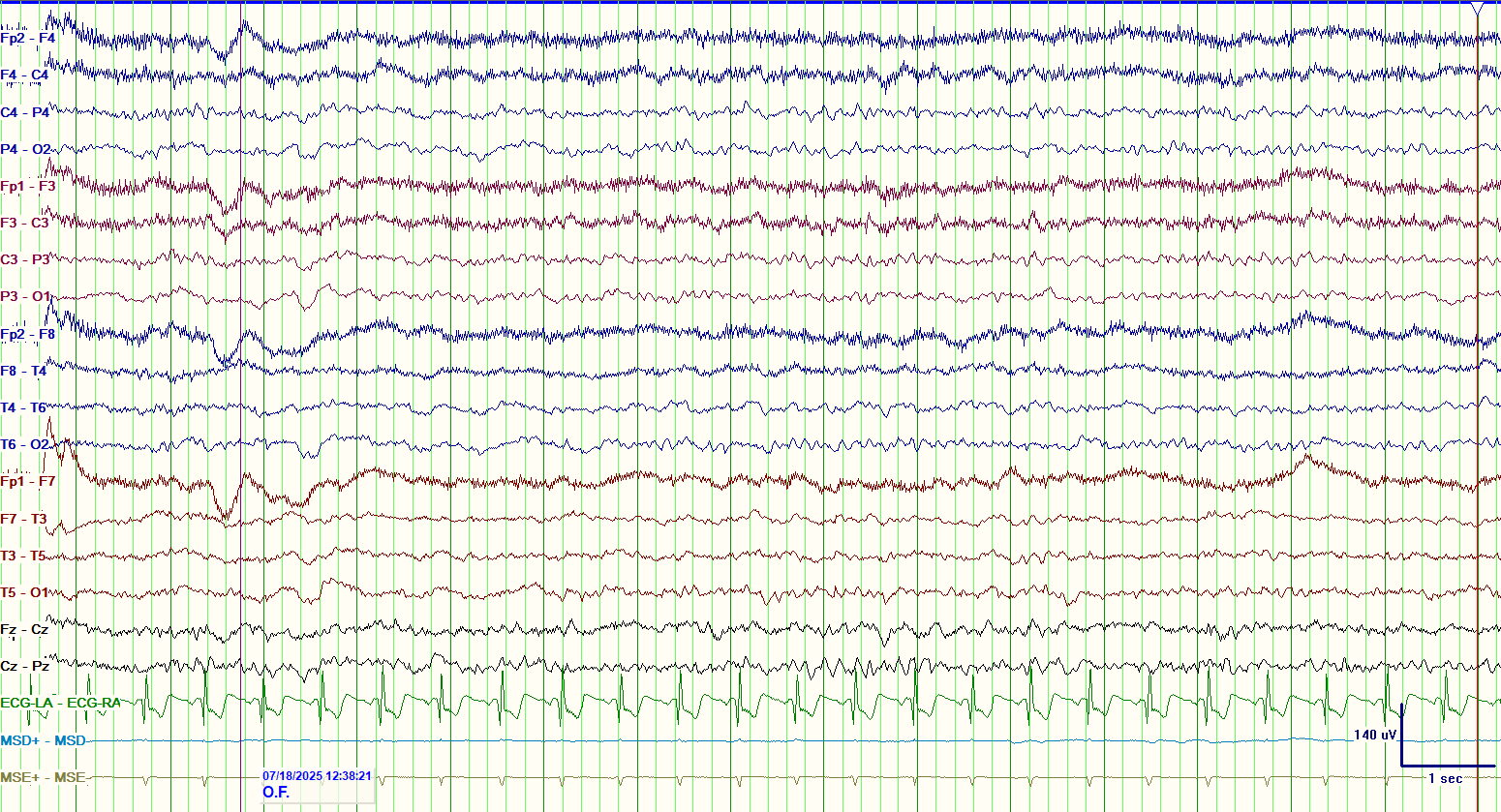


**Figure S1. Video-EEG on day 1**

International 10–20 system, bipolar montage, LFF at 1 Hz, HFF at 70 Hz, sensitivity 7 microvolts/mm.

Continuous background activity is slow and poorly organized, without a well-formed posterior alpha rhythm despite eyes being closed.

O.F - eyes closed.


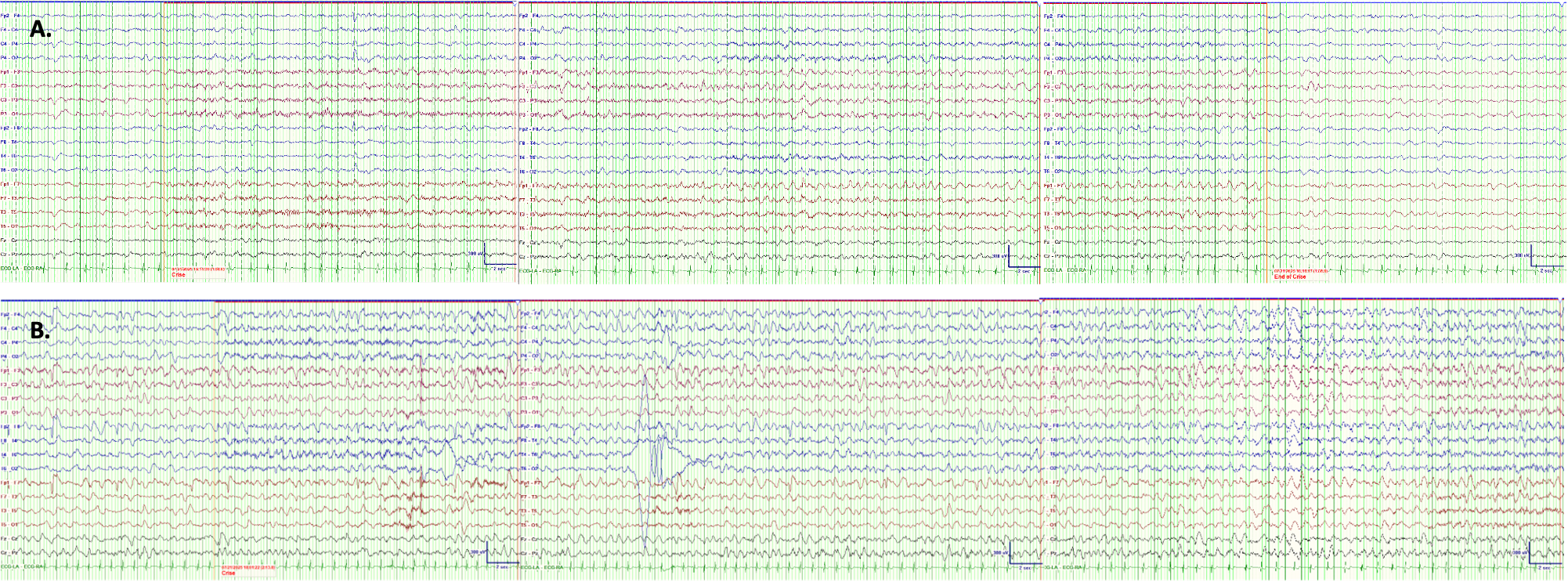


**Figure S2. Video-EEG on day 4**

International 10–20 system, bipolar montage, LFF at 1 Hz, HFF at 70 Hz, sensitivity 7 microvolts/mm.

**A.** Focal seizures originating from the left frontotemporal region, characterized by rhythmic high-amplitude discharges with progressive spatial spread. Clinically, these episodes corresponded to clonic movements of the right hemiface.

**B.** Focal seizures originating from the right hemisphere with subsequent bilateral propagation. The ictal pattern consists of rhythmic high-amplitude discharges evolving in frequency and spatial extent. Clinically, seizures were associated with rightward eye deviation, blinking, unresponsiveness, and bradypnea.


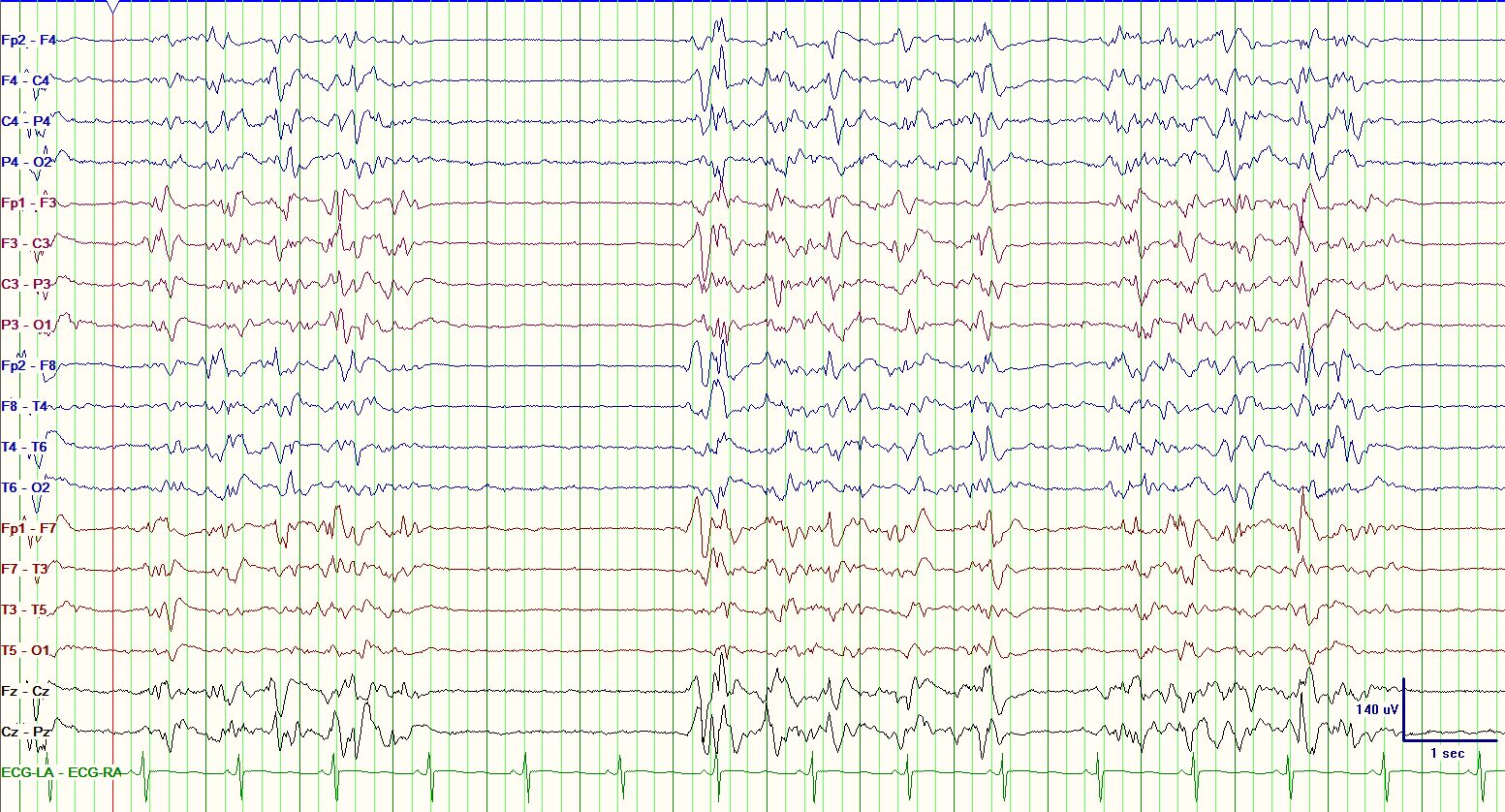


**Figure S3. Video-EEG on day 21**

International 10–20 system, bipolar montage, LFF at 1 Hz, HFF at 70 Hz, sensitivity 7 microvolts/mm.

Video-EEG shows a burst-suppression pattern characterized by alternating high-amplitude bursts and suppression phases. This recording was obtained after discontinuation of thiopental infusion, indicating persistent cortical dysfunction despite withdrawal of anesthetic sedation.


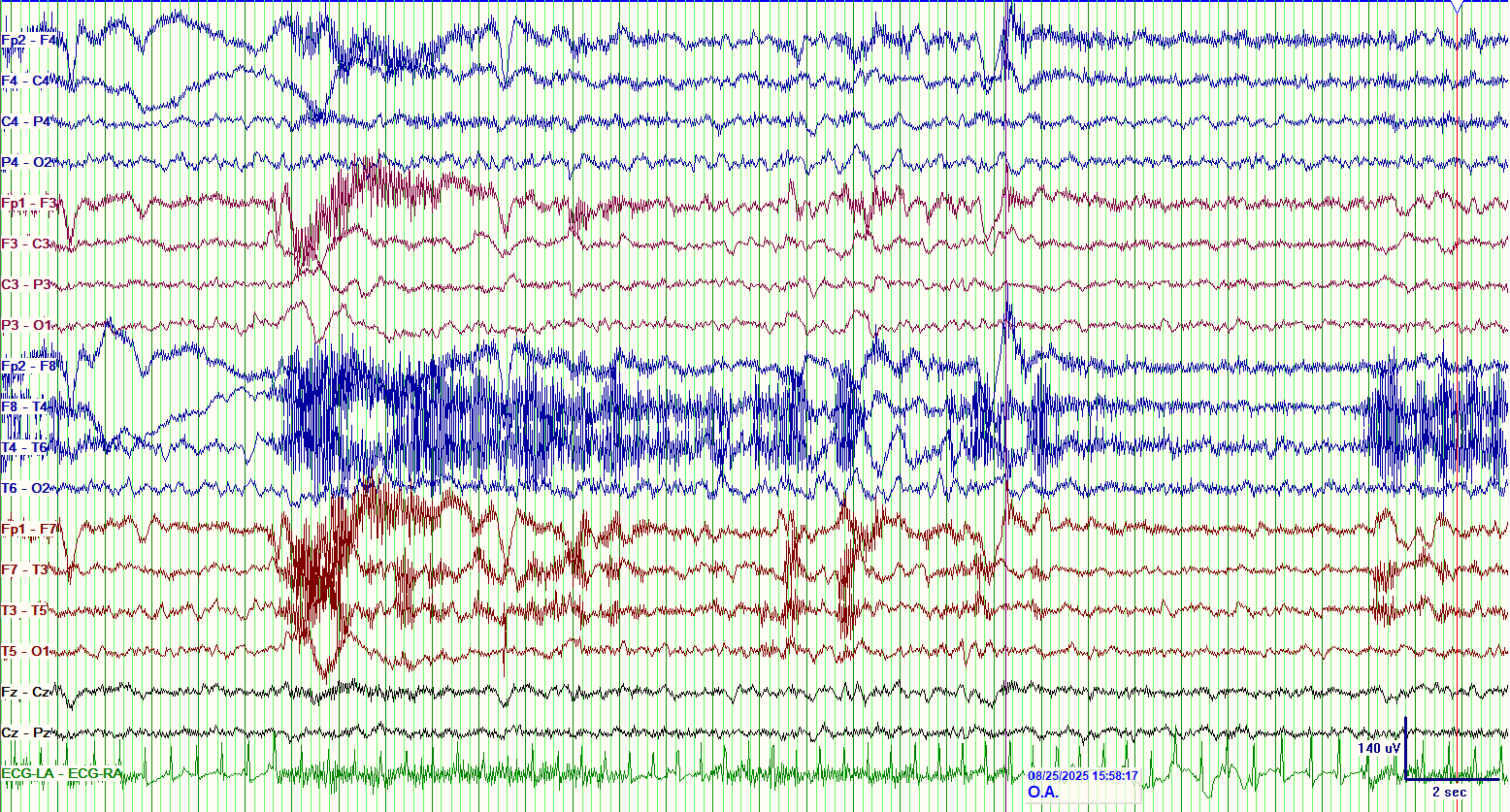


**Figure S4. Video-EEG on day 39**

International 10–20 system, bipolar montage, LFF at 1 Hz, HFF at 70 Hz, sensitivity 7 microvolts/mm.

Video-EEG recorded 24 hours after the last clinical seizure shows recovery of background organization, with a posterior rhythm of 4–5 Hz and no epileptiform activity. The recording begins with eyes closed and transitions to eyes open (O.A.). This improvement occurred after the second intrathecal dexamethasone dose.


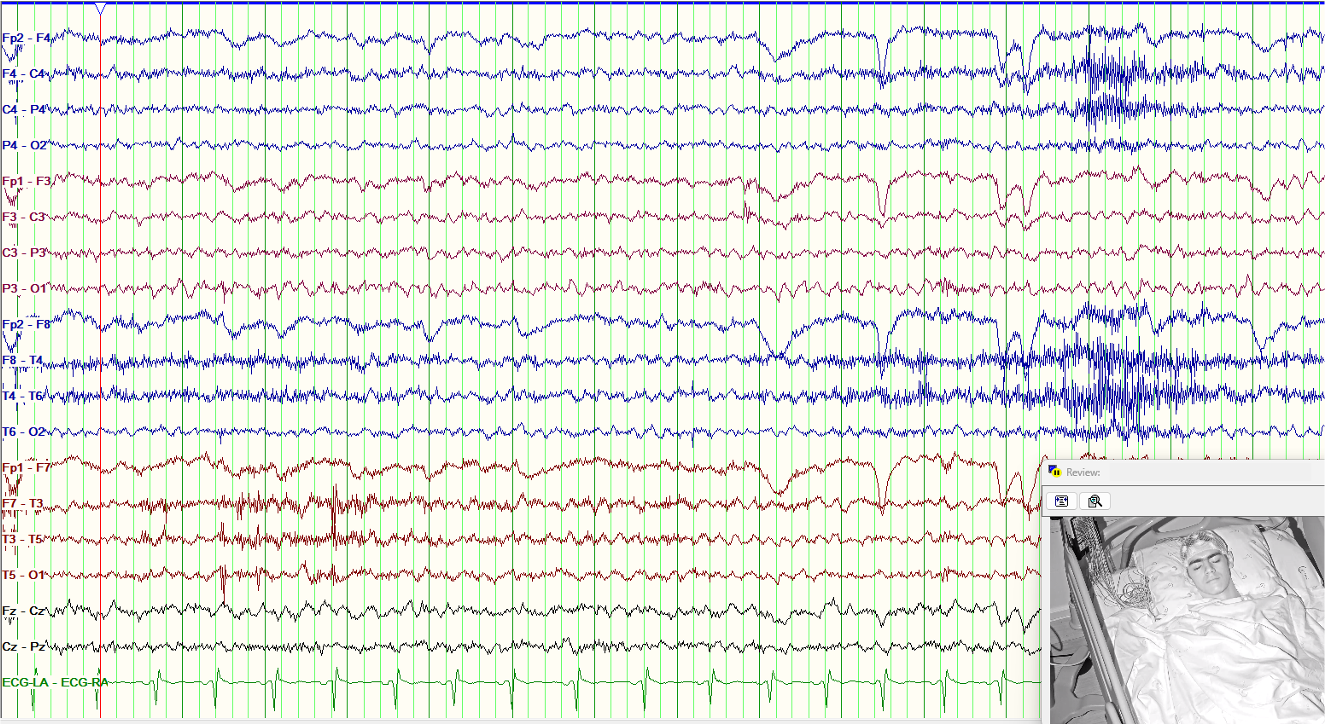


**Figure S5. Video-EEG on day 69**

International 10–20 system, bipolar montage, LFF at 1 Hz, HFF at 70 Hz, sensitivity 7 microvolts/mm.

Video-EEG recorded during wakefulness (eyes closed) shows a continuous and organized background rhythm at 6 Hz, without epileptiform activity in the captured epoch. This recording corresponds to the period of brief focal seizures recurrence in the ward, associated with febrile urinary tract infection.
